# Sequence analysis of the Epstein-Barr virus (EBV) BRLF1 gene in nasopharyngeal and gastric carcinomas

**DOI:** 10.1186/1743-422X-7-341

**Published:** 2010-11-25

**Authors:** Yuping Jia, Yun Wang, Yan Chao, Yongzheng Jing, Zhifu Sun, Bing Luo

**Affiliations:** 1 Department of Medical Microbiology, Qingdao University Medical College, Qingdao, PR China; 2 Department of Central Laboratory, Peoples Hospital of Penglai, Penglai, PR China; 3 Department of Health Sciences Research, Mayo Clinic, Rochester, Minnesota

## Abstract

**Background:**

Epstein-Barr virus (EBV) has a biphasic infection cycle consisting of a latent and a lytic replicative phase. The product of immediate-early gene BRLF1, Rta, is able to disrupt the latency phase in epithelial cells and certain B-cell lines. The protein Rta is a frequent target of the EBV-induced cytotoxic T cell response. In spite of our good understanding of this protein, little is known for the gene polymorphism of BRLF1.

**Results:**

BRLF1 gene was successfully amplified in 34 EBV-associated gastric carcinomas (EBVaGCs), 57 nasopharyngeal carcinomas (NPCs) and 28 throat washings (TWs) samples from healthy donors followed by PCR-direct sequencing. Fourteen loci were found to be affected by amino acid changes, 17 loci by silent nucleotide changes. According to the phylogenetic tree, 5 distinct subtypes of BRLF1 were identified, and 2 subtypes BR1-A and BR1-C were detected in 42.9% (51/119), 42.0% (50/119) of samples, respectively. The distribution of these 2 subtypes among 3 types of specimens was significantly different. The subtype BR1-A preferentially existed in healthy donors, while BR1-C was seen more in biopsies of NPC. A silent mutation A/G was detected in all the isolates. Among 3 functional domains, the dimerization domain of Rta showed a stably conserved sequence, while DNA binding and transactivation domains were detected to have multiple mutations. Three of 16 CTL epitopes, NAA, QKE and ERP, were affected by amino acid changes. Epitope ERP was relatively conserved; epitopes NAA and QKE harbored more mutations.

**Conclusions:**

This first detailed investigation of sequence variations in BRLF1 gene has identified 5 distinct subtypes. Two subtypes BR1-A and BR1-C are the dominant genotypes of BRLF1. The subtype BR1-C is more frequent in NPCs, while BR1-A preferentially presents in healthy donors. BR1-C may be associated with the tumorigenesis of NPC.

## Background

Epstein-Barr virus (EBV) is a ubiquitous human herpesvirus that infects over 90% of the world population. As the causal agent of infectious mononucleosis, EBV is also tightly associated with various malignancies, including Hodgkin's disease, Burkitt's lymphoma(BL), nasopharyngeal carcinoma(NPC), and B and T cell lymphomas in immunocompromised individuals such as AIDS patients and organ transplant recipients[1,2]. It is also responsible for some gastric carcinomas (GC). The EBV infection is found in 80-100% of gastric lymphoepithelioma-like carcinoma cases and 2-16% of common types of gastric adenocarcinoma [3-6]. In Northern China this rate is about 7.0% according to our previous study [7].

After primary infection, EBV establishes a lifelong, asymptomatic state in B cells. However, EBV can periodically reactivate and replicate in a lytic manner [8]. Understanding how viral latency is disrupted is a central focus in herpesvirus biology. Induction of the switch from latency to lytic cycle is associated with expression of immediate-early (IE) protein Rta (R transactivator), the product of the BRLF1 gene [9]. Rta is a 605-amino acid (AA) protein with unknown cellular homologues. The N-terminus of Rta contains an overlapping DNA binding (AA 1 to 320) and dimerization (AA 1 to 232) domain that does not correspond to any described DNA binding motif previously [10]. The transcriptional activation domain is found in the C-terminal region of the protein. An obligatory acidic activation domain (AA 520 to 605) contains highly conserved hydrophobic residues that are predicted to form alpha helices [10]. A weaker accessory activating domain contains two proline-rich subregions (AA 352 to 410 and 450 to 500).

ZEBRA, the product of EBV BZLF1 gene, had been thought to be the only viral protein capable of initiating the lytic cycle [11-14]. In recent years Rta has been found to be able to disrupt latency through activating Zp, the promoter of BZLF1, leading expression of ZEBRA, and thereby stimulation of early lytic genes, DNA replication, and late gene expression [9,15,16]. There are a number of interesting differences between the induction of lytic EBV infection by BZLF1 and that by BRLF1. Zalani S, et al. [17] reported that Rta can disrupt viral latency in an epithelial cell-specific manner (in contrast to the ability of ZEBRA to disrupt latency in B cells), and the mechanisms leading to disruption of EBV latency appear to be cell-type specific. Also, it has been demonstrated that BRLF1, not BZLF1, requires activation of the p38 and c-Jun stress MAP kinase pathways for induction of lytic EBV infection [18], and also requires PI3 kinase activation [19].

Several components of the immune system contribute to the highly efficient control of virus replication and proliferation of immortalized, EBV-infected cells in healthy individuals, and probably the most important components are HLA-restricted specific cytotoxic T lymphocytes (CTLs). As EBV can switch directly from the latent state into the lytic cycle without any expression of further latent proteins [20], CTL directed against latent proteins might not be able to prevent the ongoing viral replication. Therefore, CTL directed against immediate-early (IE) proteins is a pivotal step to control the virus lytic activation. Rta has been demonstrated to have multiple epitopes recognized by EBV-specific CTL [21,22]. Delineation of sequence variations of CTL epitopes may help the development of an effective control of EBV replication and cell proliferation.

A notable feature of EBV-associated malignancies is variation in incidence and the proportion of EBV-positive tumors in different geographic regions [23,24]. The disparity is poorly understood. To explore the potential association of the EBV-associated malignancies with integrated EBV sequence variations, as well as the possibility of a CTL-based control of EBV replication and cell proliferation, we analyzed the sequence variation of EBV BRLF1 gene in EBVaGCs, NPCs and healthy donors.

## Results

### Sequence variation of BRLF1 gene

**Figure 1:**
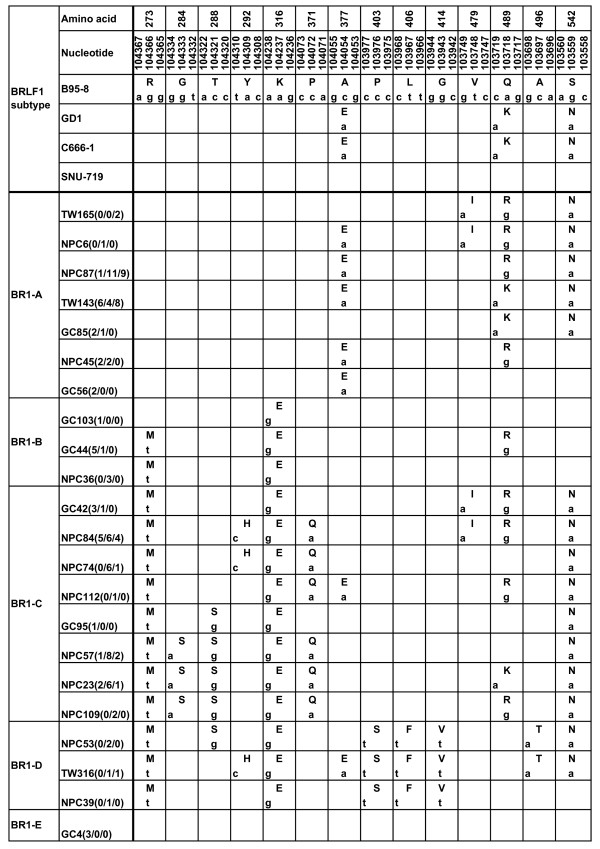
**Observed BRLF1 sequence variations in EBVaGC, NPC biopsies and TWs of healthy donors in Northern China**. The numbers in the first row correspond to the amino acid positions and the numbers in the second correspond to the nucleotide positions, under which the B95-8 prototype amino acid and nucleotide sequences are listed. Different patterns are noted to the far left column, while the specimens showing identical sequences to each other are listed by a representative isolate in the second column. The following numbers separated by "/" denote the number of the identical sequences from EBVaGC, NPC and TW, respectively. Only sequences different from B95-8 are indicated. The small letters denote the nucleotide, and the amino acids are denoted by capital letters. The GD1 sequence was taken from EBV genomes AY961628 [25]. C666-1 and SNU-719 were two EBV-positive cell lines [26,27], whose sequences were obtained by using PCR-direct sequencing method.

**Figure 2:**
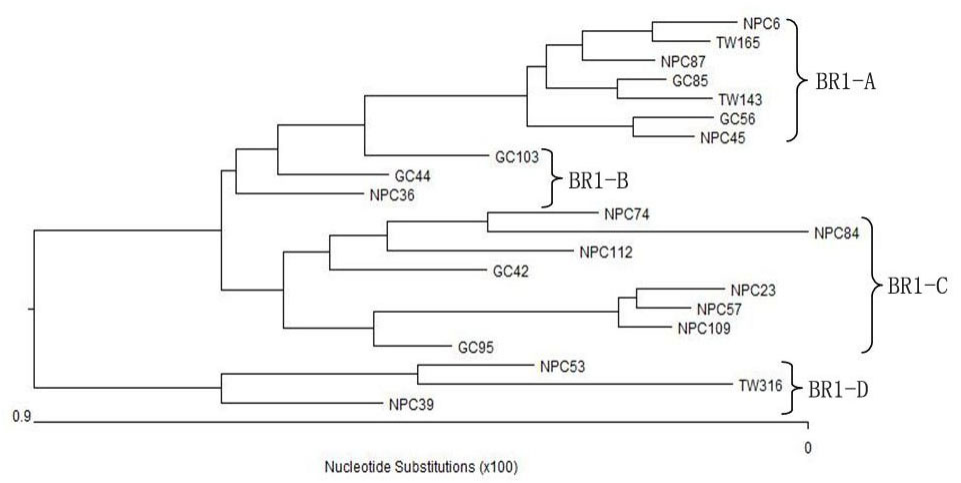
**Phylogenetic tree drawn from the BRLF1 amino acid sequences of 21 representative isolates using neighbor-joining method**. BRLF1 subtypes BR1-A, BR1-B, BR1-C and BR1-D were shown on the right. In this figure the conserved subtype BR1-E (represented by GC4) was not listed.

The sequence of BRLF1 gene coding 605 AAs was successfully amplified in 34 EBVaGCs, 57 NPCs, and 28 TWs, respectively. All the sequences were compared with the prototype B95-8 sequence. Nucleotide changes were detected in 31 loci, 14 of which resulted in AA changes. Among the 17 loci with silent nucleotide changes, one (at 103654) was detected with an A/G interchange in all the specimens tested. The translated AA mutations from the sequence variations were summarized in Figure 1. According to the phylogenetic tree (Figure 2), 5 distinct subtypes of BRLF1 were identified among the observed 119 specimens, namely subtype BR1-A, BR1-B, BR1-C, BR1-D, and BR1-E. Two subtypes, BR1-A and BR1-C, were found to be dominant in the total specimens.

The subtype BR1-A, which was represented by NPC87, was detected in 42.9% (51/119) of samples. Forty specimens in this subtype had 2 common coding changes: 377(Ala→Glu), 542(Ser→ Asn), while 6 specimens only had residue 377, 5 specimens only had residue 542 changes. Interestingly, residue 489 caused different AA changes among different specimens, namely, Gln→Arg in 28 specimens, Gln→Lys in 21 specimens. Besides these 3 residues, 3 isolates (TW165, TW121, and NPC6) showed Val→Ile interchange at residue 479. Silent changes in this pattern were detected in 3 residues: 486(CCG→CCA), 510(GAA→GAG), 572(CCC→CCA) (data not shown). The prevalent AA mutations at residues 377, 489 and 542 of this subtype were identical to the GD1 strain, which is a representative EBV strain isolated from NPC patients in Guangdong, China [25]. Also, the BRLF1 gene in C666-1 cell line, which was established from an undifferentiated NPC biopsy in Southern China [26], harbored only these three mutations.

The second common subtype BR1-C (represented by NPC57) was detected in 42.0% (50/119) of samples. This subtype contained 3 common signature residues: residue 273 (Arg→Met), 316(Lys→Glu), 542(Ser→Asn). Additionally, some isolates showed one or more additional sequence variations at other positions. Residues 284(Gly→Ser), 288(Thr→Ser), 371(Pro→Gln) coexisted in 22 specimens, while 23 specimens contained residue 371 only; one specimen (GC95) contained residue 288 only. At residue 489, 22 specimens had Gln/Arg interchange; 9 specimens had Gln/Lys interchange. An interchange Tyr/His at residue 292 was detected in 22 specimens; interchange Val/Ile at residue 479 in 19 specimens. This subtype involved 16 silent mutations in different isolates (data not shown).

The rest 3 subtypes were only detected in small numbers of specimens. The subtype BR1-B shared 2 common AA substitutions: residues 273(Arg→Met), 316(Lys→Glu). Additional Gln/Arg interchange at residue 489 was detected in 6 specimens. The subtype BR1-D was detected in 5 specimens, which had 5 common residues: 273 (Arg→Met), 316(Lys→Glu), 403(Pro→Ser), 406(Leu→Phe), 414(Gly→Val). The last subtype BR1-E, which is represented by the strain GC4, had the same AA sequence with the prototype B95-8, except for a silent mutation at 103654. This subtype was only found in 3 EBVaGCs. The BRLF1 gene in EBV-positive GC cell line SNU-719, which was established from a Korea GC patient [27], also showed conserved sequence.

### Distribution of BRLF1 subtypes in EBVaGCs, NPCs, and TWs

The frequency of BRLF1 subtypes in EBVaGCs, NPCs, and TWs of healthy donors was summarized in Table 1. Fisher's exact test was used to determine the difference of the BRLF1 subtypes among the EBVaGCs, NPCs, and the TWs. Two subtypes, BR1-A and BR1-C, were dominant in the tested specimens. BR1-A was detected in 42.9% (51/119) of total specimens, that were 13/34(38.2%) EBVaGCs, 19/57 (33.3%) NPCs, 19/28 (67.8%) TWs. BR1-C was found in 50 specimens (42.0%), including 12/34 (35.3%) EBVaGCs, 30/57(52.7%) NPCs, and 8/28 (28.6%) TWs. The present rate of BR1-A in TWs (67.8%,19/28) was significantly higher than in EBVaGCs (38.2%,13/34) or NPCs (33.3%,19/57); while, BR1-C was seen more in NPCs (52.7%,30/57) than in EBVaGCs (35.3%,12/34), or in TWs (28.6%,8/28) (*P *= 0.0019).

**Table 1:** Distribution of BRLF1 subtypes in EBVaGCs, NPCs, and TWs

BRLF1 subtypes	EBVaGC(n = 34)	NPC(n = 57)	TWs(= 28)
BR1-A	13(38.2%)	19(33.3%)	19(67.8%)
BR1-B	6(17.6%)	4(7.0%)	0
BR1-C	12(35.3%)	30(52.7%)	8(28.6%)
BR1-D	0	4(7.0%)	1(3.6%)
BR1-E	3(8.9%)	0	0

### Variation analysis in BRLF1 functional domains

As an important transactivator, BRLF1 gene harbors 3 vital functional domains: dimerization, DNA binding, and transactivation domains [10]. The AA mutations in BRLF1 functional domains were summarized in Table 2. In this study, the domain of dimerization (AA 1 to 232) was found to be stably conserved, where no AA mutations were detected (data not shown). Five residues (273, 284, 288, 292 and 316) were detected to have AA mutations in the DNA binding domain. The prevalent mutations in this domain were found in residue 273 (R→M) and 316 (K→E). The mutation R→M at residue 273 affected 50% (17/34) of EBVaGCs, 66.7% (38/57) of NPCs, 32.1% (9/28) of TW isolates; the mutation K→E at residue 316 affected 52.9%(18/34) of EBVaGCs, 66.7% (38/57) of NPCs, 32.1% (9/28) of TW isolates. The distribution of mutation R→M at residue 273 was significantly different among 3 types of specimens (*χ2 *= 9.28, *P *< 0.01; EBVaGC vs NPC: *χ2 *= 2.47, *P *> 0.05; EBVaGC vs TW: *χ2 *= 2.01, *P *> 0.05; NPC vs TW: *χ2 *= 9.05, *P *< 0.01); likewise, mutation K→E at residue 316 was distributed differently among 3 types of specimens(*χ2 *= 9.08, 0.01 <*P *< 0.05; EBVaGC vs NPC: *χ2 *= 1.70, *P *> 0.05; EBVaGC vs TW: *χ2 *= 2.70, *P *> 0.05; NPC vs TW: *χ2 *= 9.05, *P *< 0.01). In contrast, the transactivation domain was detected to have more AA mutations. Three residues, 377, 489, and 542, were the prevalent mutation loci. Mutation S→N at residue 542 affected most of the isolates (21 of 34 EBVaGCs, 50 of 57 NPCs, and all the TWs). The rest mutations in this domain affected the weaker accessory activating subregions (AA 352 to 410 and 450 to 500).

**Table 2:** Distribution of AA mutations in Rta functional domains

Functional domains	residues	EBVaGC(n = 34)	NPC(n = 57)	TWs(n = 28)
DNA binding	273(R-M)	17(50%)	38(66.7%)	9(32.1%)
	316(K-E)	18(52.9%)	38(66.7%)	9(32.1%)
Transactivation	377(A-E)	11(32.3%)	20(35.1%)	18(64.3%)
	489(Q-K)	10(29.4%)	11(19.3%)	9(32.1%)
	489(Q-R)	16(47.1%)	25(43.9%)	15(53.6%)
	542(S-N)	21(61.8%)	50(87.7%)	28(100%)

### Variation analysis of CTL epitope sequences among EBV isolates

Sixteen CTL epitopes in Rta were identified in previous studies [22,28], 3 of which showed variations in the detected isolates. Variations of CTL epitopes were summarized in Table 3. The QKE epitope was affected by an S→N change at position 14 of the epitope, and existed in the majority of the specimens (21 EBVaGCs, 50 NPCs, and 28 TWs). Mutation A→E at position three of the NAA epitope was detected in 11 EBVaGCs, 20NPCs, 18TWs, while the epitope ERP was relatively conserved; only 2 mutations P→S, L→F were detected in 4 NPCs, 1 TWs. The most common mutation S→N in QKE epitope was distributed differently in 3 sample groups (*χ2 *= 17.66, *P *< 0.01; EBVaGC vs NPC: *χ2 *= 8.37, *P *< 0.01; EBVaGC vs TW: *χ2 *= 13.55, *P *< 0.01; NPC vs TW: *χ2 *= 3.75, *P *> 0.05). Likewise, the distribution of mutation A→E in NAA epitope was significantly different (*χ2 *= 8.14, 0.01 <*P *< 0.05; EBVaGC vs NPC: *χ2 *= 0.07, *P *> 0.05; EBVaGC vs TW: *χ2 *= 6.29, 0.01 <*P *< 0.05; NPC vs TW: *χ2 *= 6.48, 0.01 <*P *< 0.05).

**Table 3:** Distribution of AA mutations in Rta CTL epitopes

HLA restriction	Rta residues	No. of isolates (%)			epitope sequence
			
		EBVaGC	NPC	TW	
B58	375-383	23(67.6)	37(64.9)	10(35.7)	N A A E P E Q P W
		11(32.4)	20(35.1)	18(64.3)	- - E - - - - - -
Cw4	393-407	34(100)	53(92.9)	27(96.4)	E R P I F P H P S K P T F L P
		0(0)	4(7.1)	1(3.6)	- - - - - - - - - - S - - F -
B61	529-543	13(38.2)	7(12.3)	0(0)	Q K E E A A I C G Q M D L S H
		21(61.8)	50(87.7)	28(100)	- - - - - - - - - - - - - N -

Sequences listed are epitope sequences of B95-8 isolate. Only the mutant AAs of the specimens are shown, while the dash indicates the identical sequence to B95-8.

## Discussion

In this study we analyzed the sequence variations of BRLF1 gene in 34 EBVaGCs, 57 NPCs and 28 TWs in healthy donors. To our knowledge, this is the first report about the polymorphism of BRLF1 gene from multiple tissues.

Based on the phylogenetic tree, we identified 5 distinct subtypes of BRLF1 gene in the specimens of Northern China. Two subtypes, BR1-A and BR1-C, were dominant in the specimens observed. In this study, subtype BR1-C was seen more in biopsies of NPC. It can be speculated that a substrain of EBV with this subtype infects NPC more frequently and this subtype may be more associated with the tumorigenesis of NPC in Northern China. Feng et al. [29] demonstrated that BRLF1 is specifically expressed in NPC tumor cells. Further studies of BRLF1 polymorphism in wider areas and functional studies of subtype BR1-C will help our understanding about the association between specific BRLF1 gene subtypes and EBV associated malignancies. Unlike subtype BR1-C, the incidence of BR1-A was significantly higher in healthy donors (67.8%) than that in EBVaGC (38.2%) or NPC group (33.3%), suggesting that this subtype was the dominant subtype of BRLF1 in healthy populations in the area studied. The prevalent mutations of this subtype were completely identical to the GD1 strain [25] and the EBV strain in NPC cell line C666-1, which were both established from Southern China. Unfortunately, we were unable to compare the prevalent rates in our samples with that in Southern China, because the distribution data of BRLF1 subtypes is not available for the populations in Southern China. Interestingly, a silent mutation A→G at 103654 was found in all the wild isolates, suggesting this interchange may be a specific marker of the EBV strains in local area and the local EBV strains may stem from a common ancestral virus different from the other BRLF1 groups.

The AA mutations in the functional domains and CTL epitopes from this study suggest that not only BRLF1 gene subtypes, but also mutations in functional domains and CTL epitopes exhibit specific distribution among 3 sample groups.

As a transcriptional activator, Rta plays an important role in the switch from latency to a productive infection. Three domains, dimerization, DNA binding, and transactivation, contribute to this function. In this study, we found the dimerization domain was highly conserved without any AA mutations, suggesting its critical function in the lytic activation. DNA binding domain was mainly affected by mutation R→M at residue 273 and K→E at residue 316 and these changes were significantly higher in NPC. R→M at residue 273 may be of great importance because interchange from hydrophilic to hydrophobic amino acid may alter the affinity of protein with DNA, while mutation at position 316 may contribute less to the change of the capacity of DNA binding, according to the results of Manet, E and colleague[10]. Although multiple AA mutations were detected in the transactivation domain, only one mutation at residue 542, which was located in the absolutely essential 90 C-terminal AAs for the protein's transcriptional activation, may have a significant impact on the transcription activity [10]. It may be of significance because it has been reported that variations in EBV-interacting molecules might alter DNA binding and transcription activity and thus may contribute to the tumorigenesis of EBV associated malignancies [30]. Interestingly, these 3 dominant mutations (R→M at residue 273, K→E at residue 316 and S→N at residue 542) affecting functional domains were all included in the subtype BR1-C, but the two mutations at residues 273 and 316 were not detected in the isolates of subtype BR1-A at all (0/119). The subtype BR1-A is preferentially present in healthy donors, while BR1-C is more frequent in NPCs. Moreover, the two mutations at residues 273 and 316 in BR1-C were seen more in NPC. These observations indicate that these two AA mutations may be of great importance in the carcinogenesis of NPC. These two mutations can also be potentially used as a gene marker to distinguish subtype BR1-A from other subtypes in the area observed.

Studies have shown that EBV can elicit strong CTL responses which direct against a limited number of viral proteins [31-33]. Focus has been on the mutations of CTL epitopes in EBV latent-expressing proteins for their important roles in the associated malignancies, while little is known about the proteins which are expressed in the lytic phase. In the present study, we found 3 of 16 identified CTL epitopes of Rta were affected by AA mutations (Table 3). Epitope ERP was affected in a few isolates; while epitopes NAA and QKE were frequently affected by mutations, with relatively lower mutation rates in malignant groups (EBVaGC or NPC) than in healthy donors. This was contrary to the general belief that the viral strains associated with malignancies can evade immune surveillance by altering amino acids within CTL epitopes [34]. CTL directed against immediate-early (IE) proteins is a pivotal step to control the virus lytic activation. The sequence analysis to all known Rta CTL epitopes provides valuable information for choosing target epitopes for control of EBV lytic activation.

## Conclusions

In conclusion, we have identified 5 distinct subtypes of BRLF1 in Northern Chinese EBV isolates in multiple clinical specimens. The subtype BR1-C is more frequent in NPCs, while BR1-A preferentially presents in healthy donors. Mutation analysis in functional domains and CTL epitopes revealed specific distribution of mutations among 3 specimen groups. The impact of these alterations on functions of Rta and immunological recognition of EBV is potentially interesting and needs more functional studies. Further investigation in extended areas and EBV associated diseases will enhance our understanding of BRLF1 gene polymorphism and their association with tumors.

## Materials and methods

### Specimens, cells and DNA extraction

Thirty-four EBVaGCs, 57 NPCs, 28 TWs and 2 EBV positive cell lines (GC cell line SNU-719, NPC cell line C666-1) were used in this study. Tumor tissues of GCs and NPCs were collected from major hospitals of Shandong Province in the Northern China, a non-endemic area of NPC. The infection of EBV in GC and NPC tissues was determined by EBV-encoded small RNA (EBER) 1 in situ hybridization, as described previously [35]. TWs were collected from the healthy donors in the same geographic regions. The EBV-positive TWs were determined by the BamHI W fragment positive signals, using PCR with a BamHI W specific primer pair [36]. EBV positive cell lines SNU-719 and C666-1 were maintained in RPMI 1640 supplemented with 10% fetal bovine serum (FBS) [26,27]. The B95-8 cell line was used as a source of the prototype EBV genome. All the carcinoma patients as well as the healthy individuals gave an informed consent for the study and the study was approved by the Medical Ethics Committee at the Medical College of Qingdao University, China.

DNAs used in this study were extracted from fresh specimens and cell lines by using the standard method with proteinase K digestion and phenol-chloroform purification. QIAamp DNA FFPE Tissue kit (QIAGEN GmbH, Hilden, Germany) was used to extract the DNA from paraffin-embedded tumor tissues.

### Amplification of DNA

Specific oligonucleotide primers flanking the BRLF1 gene were designed for nested PCR (Table 4). In each set of PCR, DNA from EBV-positive B95-8 cell lines was used as positive control, and nuclease-free distilled water served as negative control. For the amplification, the first round polymerase chain reaction (PCR) was performed in a total volume of 25 μl containing 1 × PCR reaction buffer, 100 ng of genomic DNA, 0.5 μM of each primer, 200 μM of each deoxyribonucleotide triphosphates, and 1 U Pfu Taq polymerase (TaKaRa Biotechnology Co., Ltd., Kyoto, Japan). PCR amplification was performed with an initial denaturation at 95°C for 5 min. Then, 35 cycles of denaturation at 94°C for 30 s, annealing at 55°C for 30 s, extension at 72°C for 1 min. A final elongation step at 72°C for 10 min was also conducted. BRLF1-A1 combined with BRLF1-A2 (splice1), BRLF1-B1 with BRLF1-B2 (splice2) and BRLF1-C1 with BRLF1-C2 (splice3) as the outer primers. When necessary, 2 μl of the PCR product were taken for a second round of PCR, using internal primers: BRLF1-A2 combined with BRLF1-A3 (splice1), BRLF1-B3 with BRLF1-B4 (splice2), and BRLF1-C3 with C4 (splice3). In order to prevent contamination, several measurements were taken, such as frequently changing gloves and cleaning the equipment, using aerosol-resistant pipette tips for PCR, and performing different procedures in separate areas. The PCR products were analyzed by electrophoresis through a 1.2% agarose gel.

**Table 4:** Sequence and coordinates of primers used in PCR and sequencing

Primers	sequence(5'-3')	B95-8 coordinates
Splice1		
BRLF1-A1	GGTGCAATGTTTAGTGAGTTAC	103187 - 103208
BRLF1-A2	ACCAAGAGAGCGATGAGAGA	104021 - 104002
BRLF1-A3	GGAGGCAGTTTTCAGAAGTGT	103345 - 103365
Splice2		
BRLF1-B1	TTTGGCTGACACACCTCTCG	103847 - 103866
BRLF1-B2	CCACCATAGGCACCGCTATG	104783 - 104764
BRLF1-B3	CATACCTTCCCGGCTATCCCT	103918 - 103938
BRLF1-B4	GTGTTCACCTATCCCGTCCTC	104598 - 104578
Splice3		
BRLF1-C1	ACTTGGTTGACAGCAGGCA	104409 - 104427
BRLF1-C2	GGTGGCTAGGTGGGAGGT	105323 - 105306
BRLF1-C3	CAGAGCCCTGACATCCTTA	104503 - 104521
BRLF1-C4	CACCACATCCCCCACTTC	105274 - 105257

### Sequencing analysis of PCR products

PCR products were purified using a gel extraction kit (QIAEX II; QIAGEN GmbH, Hilden, Germany), under the conditions specified by the manufacturer. PCR amplified fragments were sequenced by means of a Prism ready reaction Dyedeoxy terminator cycle sequencing kit (Applied Biosystems, Foster, USA).

### Data analysis

The sequence data were checked for any homology in BLAST (National Center for Biotechnology Information; http://www.ncbi.nlm.nih.gov/) and were compared with the B95-8 prototype strain. Alignments between sequences were analyzed using DNA Star software (DNASTAR, Inc, version 5.0). The sequences from representative samples were used to draw a phylogenetic tree. Either χ2 test or Fisher's exact test (2-sided) was performed to determine the distribution difference of the EBV variations among the EBVaGCs, NPCs, and the TWs from the healthy adults. Significance was set at *P *value < 0.05.

## Competing interests

The authors declare that they have no competing interests.

## Authors' contributions

YPJ carried out most of the studies and drafted the manuscript. YW and YC participated parts of the studies and writing. YZJ was responsible for the collection of specimens used in this study. ZFS and BL provided consultation and preparation of the final report. All authors read and approved the final manuscript.
